# New York Odontological Society

**Published:** 1884-05

**Authors:** 


### NEW YORK ODONTOLOGICAL SOCIETY.

This Society held its March session at the house of Dr. Bodecker, on the evening of the 18th ult., the president, Dr. Jarvie, occupying the chair.

A plaster model was exhibited, representing the mouth of a child eight years of age, with a misshaped central incisor, somewhat dwarfed, pitted, and out of proper position. Opinions solicited brought out several responses, all to one effect—to "leave it undisturbed for the present," or, according to Micawber, "to wait and see what may turn up." The "misshaped" tooth was apparently an intrusive supernumerary.

*Dr. C. F. Parker*—Exhibited beautiful specimens of gold crowns, swaged from a single plate of gold with metal dies.

*Dr. N. W. Kingsley*—Considered them faulty, inasmuch as crowns so made could not be fitted accurately to the cervical parts of teeth or roots. Dr. K. explained his method of making gold crowns, first fitting a ferrule-like band around the tooth to be enclosed, taking care to have it extend beneath the border of the gum, and clasping the tooth snugly. With this band adjusted, and the open part filled with wax, a "bite," or impression, is taken of the grinding surface of its occluding companion, then a cast poured, from which a die is made for swaging or punching out a cap, which is fitted and soldered to the band. The completed crown is secured to the natural organ with a stopping of oxy-phosphate of zinc. A small hole should be drilled through the gold crown before the final adjustment, to permit the excess of filling to escape.

*Dr. S. G. Perry*—Makes similar operations, but forms his crowns of platinum.

*Dr. E. A. Bogue*—Asked if any of the gentlemen present had used gutta-percha stopping for securing caps or crowns. He had much confidence in this material.

*Dr. C. E. Francis*—Stated that he had frequently used gutta percha for this purpose, and related an instance where he secured a gold crown to a fractional part of a bicuspid over twenty-five years ago, and which did good service for eight years. A severe lateral strain finally caused the tooth-fragment to break, but the dentine and stopping were both white and clean. Dr. F. stated also that Dr. Kingsley had described in detail his own method of making and setting gold crowns; and of the many he had adjusted, not one, so far, had failed or disappointed him.

*Prof. Frank Abbott*—Remarked that gold crowns had been used for over a quarter of a century, by Drs. Dwinelle, Francis, and others, as shown by records, etc., and, consequently, were "nothing new."

The President introduced Prof. Chas. Mayr, of Springfield, Mass., who gave a dissertation on the "Chemistry of Antiseptics and Disinfectants." Drawing an explicit line of distinction between these agents, he dilated broadly upon each. "Disinfectants," said he, "by chemical action destroy odors, while antiseptics destroy agents that produce odors. The best disinfectants are those that impart the greatest amount of oxygen, the bromides coming first on the list. The timely use of antiseptics would render the use of disinfectants unnecessary. In the treatment of diseased teeth, three objects are to be sought: First, to destroy and remove the infusorial germs; second, to embalm any germs still remaining; third, to keep out a new stock of infusoriæ. The best antiseptic, and surest agent for killing the germs, is bichloride of mercury. This, however, is a heroic agent, which destroys not only germs, but tissue, hence must be used with great care. It kills bacteria instantly at a strength of $\frac{1}{2000}$. The cavities can be bathed with this solution, but afterwards should be well washed out with water. After destroying the germinal matter, the embalming process may follow. Carbolic and salicylic acid, eucalyptus, ol. terebinth, and some essential oils, are used for this purpose." Prof. M. explained the properties and action of several of these disinfectants, classing carbolic, kresylic, and phenic acids, and creosote as members of the same family.

"After the embalming process, the cavity must be so securely sealed that nothing can enter, for if the slightest opening is left the infusoriæ will sail in. Bacteria will follow wherever moisture can penetrate. They will enter a crevice of $\frac{1}{30000}$ of an inch.

"The enamel is sufficiently compact and dense to keep out putrefactive elements, but the exposed dentine is no barrier to their ravages. Arsenic is not antiseptic. Vessels in which it is kept, and their stoppers, gather coatings of mould. Arsenic favors putrefaction. A compound of boracic acid with glycerine forms an excellent antiseptic. Without living organisms there can be no putrefaction. Bacteria are found in healthy blood. There are no antiseptics that equal the mercurials. Iodides, iodoform, etc., and the bromides, emit unpleasant odors, and hence are objectionable. It is the same with turpentine. This acts only from the oxygen it imparts. Kresylic acid, as a disinfectant, is longer retained in the cavity than most other agents, consequently is better. Disinfectants are purely chemical in action."

In response to an inquiry by Dr. Atkinson, as to the difference between "fermentation" and "putrefaction," Prof. M. stated that "fermentation is the breaking up of substances not containing albumen or nitrogen; while putrefaction breaks up substances containing albumen, nitrogen, etc. One acts on vegetable product, and the other on animal tissue."

